# Energy Transport along *α*-Helix Protein Chains: External Drives and Multifractal Analysis

**DOI:** 10.3390/ma15082779

**Published:** 2022-04-10

**Authors:** Narmin Sefidkar, Samira Fathizadeh, Fatemeh Nemati, Constantinos Simserides

**Affiliations:** 1 Department of Physics, Urmia University of Technology, Urmia 5716693187, Iran; narmin.sefidkar@gmail.com (N.S.); fatemeh.nemati.1988@gmail.com (F.N.); 2 Department of Physics, National and Kapodistrian University of Athens, Panepistimiopolis, Zografos, GR-15784 Athens, Greece; csimseri@phys.uoa.gr

**Keywords:** energy transport, protein chain, temperature, salt concentration, external mechanical drives, molecular motor

## Abstract

Energy transport within biological systems is critical for biological functions in living cells and for technological applications in molecular motors. Biological systems have very complex dynamics supporting a large number of biochemical and biophysical processes. In the current work, we study the energy transport along protein chains. We examine the influence of different factors such as temperature, salt concentration, and external mechanical drive on the energy flux through protein chains. We obtain that energy fluctuations around the average value for short chains are greater than for longer chains. In addition, the external mechanical load is the most effective agent on bioenergy transport along the studied protein systems. Our results can help design a functional nano-scaled molecular motor based on energy transport along protein chains.

## 1. Introduction

Manipulating nature’s machinery at small length scales is an exciting opportunity for biologists, chemists, and physicists. Developing our knowledge of biological patterns enables us to create real single-molecule devices for technological applications. Due to already accomplished research, biological molecular motors such as kinesin or dynein can be used to design and develop nano-scale machines [1,2]. According to the conservation of energy viewpoint, such molecular motors take the energy produced during adenosine triphosphate (ATP) hydrolysis and dissipate it as heat in the surrounding water. However, the energy released during ATP hydrolysis does not dissipate among the numerous degrees of freedom of water instantly. The energy is “conserved” in some form attributed with one or a few degrees of freedom, for a “long” period of time, up to a millisecond, allowing the molecular motor to perform mechanical work. Therefore, bio-energy and its transfer or transport are essential issues in living systems due to their relation to biological processes and molecular motor functionality.

According to biochemical investigations, bio-energy is produced due to the hydrolysis reaction of ATP. The energy released in the hydrolysis of an ATP molecule is ≈0.43 eV under normal physiological conditions [3,4,5]. Specifically, the transformation of ATP to ADP (adenosine triphosphate) follows the famous reaction ATP${}^{4-}$ + H${}_{2}$O → ADP${}^{3-}$ + HPO${}_{4}^{2-}$ + H${}^{+}$ + 0.43 eV. ATP molecules are found primarily in the cell’s mitochondrion and confined to a particular locus on the protein chain. There is a bio-energy mobility task from a position where energy is produced to the tissues that require energy via protein molecules. As a result, we can state that bio-energy transport is carried out through protein biomolecules. Energy transport along proteins, contemplated as molecular machines for their work and function, is surveyed in two aspects: propagation of conformational changes and elimination of extra heat [6].

The two protein structural elements, the α-helices and the β-sheets, are stabilized by hydrogen bonds. Since helices span the entire protein, it was guessed that they transmit vibrational energy through the biomolecule [7]. Experimental studies propose that in ATP hydrolysis, ≈0.205 eV (half of the released energy) appears in the amide-I (C=O) bond vibration. This effect is called self-trapping of the amide-I vibrational quantum [4]. This indicates that the energy is coupled with other degrees of freedom and involves quantum effects. The released energy could change the protein’s conformation and configuration via the amide-I vibration. According to Pang [4], Davydov [7,8,9] “took into account the coupling between the amide-I vibration (intramolecular excitation or “exciton”) and deformation of amino acid residues (or, acoustic phonon) in the α-helix proteins and gave further the Hamiltonian of the system”, which has three parts, one for “excitons”, one for “phonons”, and one for their interaction (cf. Equation (1)). The term “exciton” here is not to be confused with optical excitations involving an electron coupled with a hole; it means the excitation of vibrations in the C=O bond. In Ref. [8], titled “The theory of contraction of proteins under their excitation”, Davydov epitomizes: “the quantum theory of contraction of α-helical proteins under the excitation of their peptide groups, which form three parallel chains of hydrogen bonds along the molecule. In the region embraced by an excitation, the pitch of the helix decreases, and the ⟪contracted⟫ region of the helix moves along the molecule at a rate proportional to the energy of resonance interaction between peptide groups.” The resonance (or dipole–dipole) interaction between adjacent C=O bonds allows vibration energy to be transmitted through the polypeptide chains. These vibrations result in distortion of the amino acid residues, which are combined with the amide-I vibrational quanta via acoustic phonon—“exciton” coupling that forms solitons. Some theoretical works focus on bio-energy transfer in protein α-helical structures, such as those of Davydov [3,8], Takeno [10], Yomosa [11,12], and Pang [4,5,13,14]. Bio-energy transport along protein molecules is a complex process. Its fundamental physics concerns nonlinear interactions, phonons, protein molecule deformation, solitons, and mutual interactions between them. In the α-helix protein structure, the exciton system model describes how the nonlinear interaction of the vibrational exciton and the deformation leads to self-trapping of the amide-I oscillations, restricting their dispersion. The polaronic self-trapped vibrational energy, along with local protein structural deformation, can propagate as a traveling pulse along the chains, which are known as the spines of the α-helix [15].

Davydov formulated a reasonable concept about how the protein might preserve some energy from rapid equipartition (thermalization) based on the quantum self-trapping of exciton [9,16,17]. Davydov considered α-helix as a crystal wherein the excitation of the amid bonding is anharmonic, since it is related to the helix’s elastic modes (phonons) by hydrogen bonds. Such interaction results in the stable presence of a specific quasi-particle that may quantum mechanically migrate along the α-helix with no apparent dissipation. In the current study, we have used a modified Davydov model to investigate the energy transport in α-helix protein chains for the design and control of a biomolecular motor. It is difficult to control the function of such devices in biological systems under changing conditions. We have used different agents such as the environmental temperature, the solvent parameter, and application of an external mechanical force to modulate the energy transport in a protein chain and to control the motor function. This has numerous potential applications in nanotechnology.

The rest of the article is organized as follows. In Section 2, we describe our model and methods, in Section 3, we present our results for the effect of length (Section 3.1), temperature (Section 3.2), solvent (Section 3.3), and external force (Section 3.4) as well as multifractal analysis (Section 3.5). We continue with the discussion in Section 4, and in Section 5, we state our conclusions.

## 2. Model and Methods

We consider a polypeptide chain arranged in a one-dimensional conformation of the helical structure of the protein. The α-helix is a common element of the protein secondary structure, which is formed when amino acids wind up to form a helix, where the side chains point out from the central coil. The α-helix is a rod-like structure whose inner section is formed by a tightly coiled main chain, with its side chains extending outward in a helical array. The alpha helix structure takes advantage of the hydrogen bond between CO and NH groups of the main chain to stabilize. In the optimized Davydov model (Pang model), the Hamiltonian of the protein chain is defined as follows [5,18]:

(1) $$H=H_{\mathrm{ex}}+H_{\mathrm{ph}}+H_{\mathrm{ex}-\mathrm{ph}}.$$

$H_{\mathrm{ex}}$ is the part describing the kinetic energy of the exciton and the resonant (dipole-dipole) interaction between adjacent excitons in the protein lattice,

(2) $$H_{\mathrm{ex}}=\sum_{i}\left[\varepsilon_{0}B_{i}^{†}B_{i}-J\left(B_{i}^{†}B_{i+1}+B_{i+1}^{†}B_{i}\right)\right],$$

where $\varepsilon_{0}=0.205$ eV is the exciton energy, $B_{i}^{†}\left(B_{i}\right)$ is the creation (annihilation) operator of an exciton in site *i*, and $J=9.68\times 10^{-4}$ eV is the dipole–dipole interaction energy between adjacent sites [4,18,19].

$H_{\mathrm{ph}}$ is the part defining the deformation of amino acid residues (acoustic phonon) in the protein system,

(3) $$H_{\mathrm{ph}}=\sum_{i}\left[\frac{P_{i}^{2}}{2m}+\frac{1}{2}\omega {\left(u_{i}-u_{i-1}\right)}^{2}\right],$$

where $u_{i}$ and $P_{i}$ are the displacement of the peptide groups and the conjugate momentum operator, respectively. m= [1.17–1.91] $\times 10^{-25}$ kg = [70–115] amu is the mass of amino acid residue in α-helix proteins, ω= [13–19.5] N/m = [0.81–2.37] eV/Å${}^{2}$ is the elastic constant of the protein molecular chains for α-helix proteins [4].

The interactions between the intramolecular excitation and displacement of adjacent amino acids can be described in $H_{\mathrm{ex}-\mathrm{ph}}$ as

(4) $$H_{\mathrm{ex}-\mathrm{ph}}=\sum_{i}\left[\chi_{1}\left(u_{i+1}-u_{i-1}\right)B_{i}^{†}B_{i}+\chi_{2}\left(u_{i+1}-u_{i}\right)\left(B_{i}^{†}B_{i+1}+B_{i+1}^{†}B_{i}\right)\right],$$

where $\chi_{1}=62$ pN = 38.7 meV/Å and $\chi_{2}=$[10–18] pN = [6.24–11.23] meV/Å are coupling constants that modulate the on-site energy and resonant interaction energy for the excitons caused by the molecular displacements, respectively [4].

We have derived the evolution equations of the system directly from the Hamilton equation $\left(\dot{p_{i}}=-\frac{\partial H}{\partial q_{i}}\right)$ and Heisenberg equation $\left(\dot{B_{i}}=-\frac{i}{\hbar }=\left[B_{i},H\right]\right)$, where $p_{i}$ and $q_{i}$ are the generalized momentum and coordinate, respectively. Specifically,

(5) $$\begin{array}{ccc} m{\ddot{u}}_{i} & = & \omega \left(u_{i+1}+u_{i-1}-2u_{i}\right)+\chi_{1}\left(\right|B_{i+1}{|}^{2}-|B_{i-1}{|}^{2})\\ & + & \chi_{2}\left(B_{i}^{†}B_{i+1}+B_{i+1}^{†}B_{i}-B_{i}^{†}B_{i-1}-B_{i-1}^{†}B_{i}\right), \end{array}$$

and

(6) $$\begin{array}{ccc} i\hbar {\dot{B}}_{i} & = & \varepsilon_{0}B_{i}-J\left(B_{i+1}+B_{i-1}\right)+\chi_{1}\left(u_{i+1}-u_{i-1}\right)B_{i}\\ & + & \chi_{2}\left[\left(u_{i+1}-u_{i}\right)B_{i+1}+\left(u_{i}-u_{i-1}\right)B_{i-1}\right]. \end{array}$$

We have directly derived the exciton energy transport rate from the continuity equation for the exciton density, as

(7) $$j_{i+1}-j_{i}=\frac{dn_{i}}{dt},$$

where $j_{i}$ is the local exciton flux and $n_{i}=B_{i}^{†}B_{i}$ is the exciton density. The energy transport rate operator $j\left(t\right)=\sum_{i}j_{i}$ is defined as

(8) $$\begin{array}{ccc} j(t) & =\frac{i}{\hbar } & \sum_{i}[J\left(B_{i+1}^{†}B_{i}-B_{i-1}^{†}B_{i}\right)\\ & - & \chi_{2}\left(u_{i+1}-u_{i}\right)B_{i+1}^{†}B_{i}+\chi_{2}\left(u_{i}-u_{i-1}\right)B_{i-1}^{†}B_{i}]. \end{array}$$

## 3. Results

Energy transport is a challenging problem in biological processes and biotechnological devices. In this regard, we have investigated some crucial factors of the energy transport through protein chains. Specifically, we investigate the influence of length, temperature and temperature gradient, solvent, and external force magnitude and frequency. Finally, we perform a multifractal analysis.

### 3.1. Length

**Figure 1:**
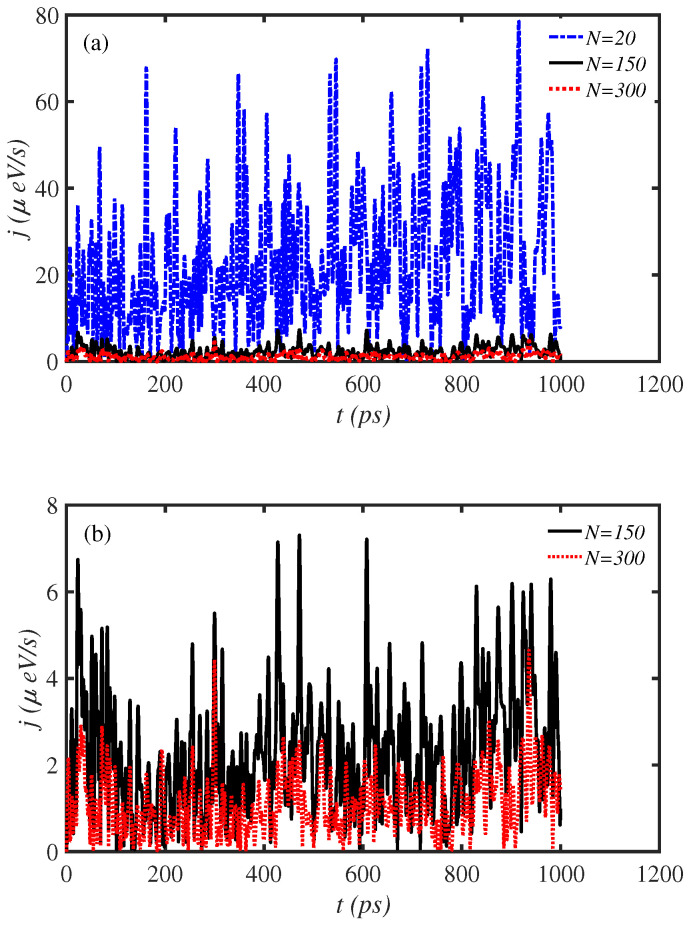
Time series of energy transport rate, *j*, along protein chains of different length. (**a**) The blue dashed line, the black solid line, and the red dotted line correspond to N=20, N=150, and N=300 site amino acid chains, respectively. (**b**) The black solid line and the red dotted line correspond to N=150 and N=300 site amino acid chains, respectively.

The length of a biological dynamical system can be an influential factor on energy transport along it. Therefore, we have investigated the energy transport along proteins with different amino acid numbers (cf. Figure 1).

Figure 1 shows the time series of exciton energy transport rate (*j*) for protein chains with different amino acid numbers. The exciton energy fluctuates in time for all three lengths of the protein chain. However, the amplitude of these fluctuations is different for different sizes. The amplitude of energy fluctuations decreases through the increase in chain length. The average value of the exciton energy transport rate for N=20 amino acid sites is $j_{ave}=23.2\;\mu$eV/s, and for N=150 and N=300 amino acid sites are $j_{ave}=2.30\;\mu$eV/s and $j_{ave}=1.12\;\mu$eV/s, respectively. The exciton energy transport rate fluctuates around the average value. The fluctuations around the average value for the smaller chain are greater than for the other chains. The amplitude of energy fluctuations reaches up to 80μeV/s for a chain of N=20 amino acid sites (Figure 1a), while the amplitude of energy fluctuations is up to 5μeV/s for a chain of N=300 amino acid sites (Figure 1b). The results are obtained at physiological temperature T=300 K.

### 3.2. Temperature

Temperature has an essential effect on the stability of solitons excited in protein chains. The effect of temperature on the solitons has been studied in many models of the protein molecules [13,20]. These studies showed that the temperature of the environment causes anharmonic vibrations of amino acid residues or peptide groups and excitations of thermal phonons in such systems.

In this regard, we model the influence of environmental temperature as a thermal bath. In other words, we have immersed the system in a thermal bath. In this regard, a Nosé–Hoover thermostat as a temperature source was chosen; its temperature can be fixed at desired temperatures. The evolution equation of the thermodynamics friction coefficient, which interacts with the system sites in the Nosé–Hoover thermostat, is given by [21,22]

(9) $$\dot{\xi }=\frac{1}{M}\left[\sum_{i}m{\dot{u}}_{n}^{2}-Nk_{B}T\right],$$

where $k_{B}$ is the Boltzmann’s constant, *T* is the temperature, and M=1000 is the thermostat constant. On the other hand, the evolution equation ${\dot{u}}_{i}$ is corrected by the $-u_{i}\xi$ term.

**Figure 2:**
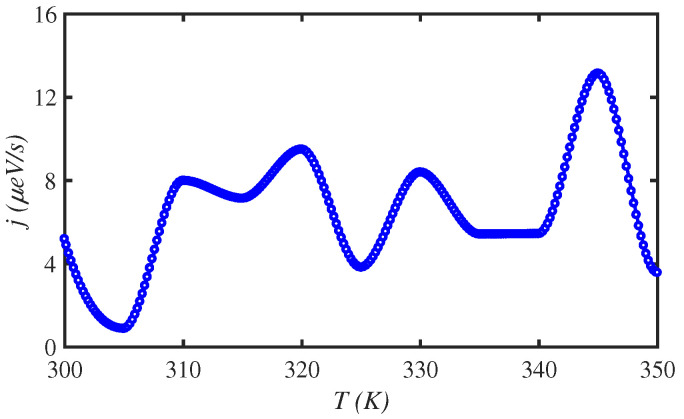
The energy transport rate, *j*, along a N=150 site amino acid chain, for different temperatures, *T*.

Figure 2 shows the fluctuation of energy transport rate of a N=150-site amino acid chain for different temperatures. We have changed the temperature from physiological temperature T=300 K up to T=350 K. It is shown that the temperature as a critical factor affects the energy transport rate of the system. The energy transport rate reaches j=13μeV/s at T=345 K. Thus, it is crucial that one can modulate the temperature to obtain the helpful energy for biological or technological applications.

**Figure 3:**
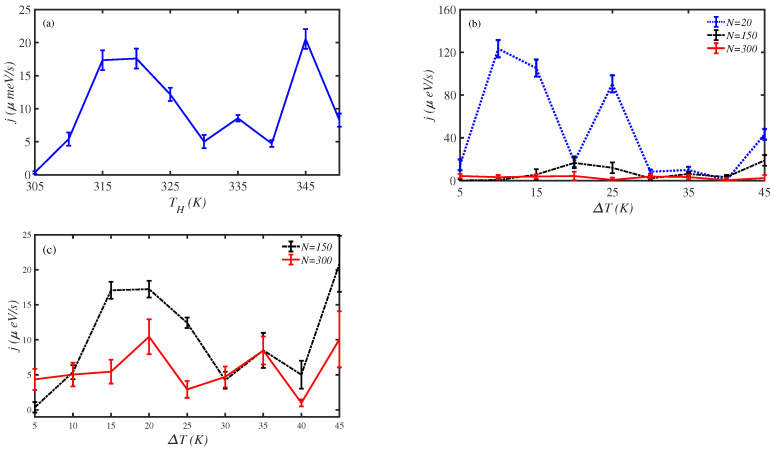
The energy transport rate, *j*, along a N=150 site chain, (**a**) for different temperatures of hot source, $T_{H}$, at a fixed temperature difference, ΔT=5 K, and (**b**) for different temperature differences, ΔT, and the temperature of the cold source, $T_{C}=$ 300 K. (**c**) The zoomed figure of the energy transport rate, *j*, along the N=150 and N=300 site chains for different temperature differences, ΔT.

In the following, we have examined the effect of temperature difference between the two ends of the protein chain. At first, we have fixed the temperature difference at ΔT=5 K and varied the temperature of the two ends. Figure 3a shows the fluctuations of the energy transport rate of a N=150-site amino acid chain for a different hot source temperature, $T_{H}$. We obtain that a maximum energy transport rate, j=20μeV/s, occurs at $T_{H}=345$ K, where the cold source temperature is $T_{C}=340$ K.

Secondly, we have fixed the temperature of the cold end at $T_{C}=300$ K and changed the temperature of the hot end from 305 K up to 350 K. This means that the temperature difference between the two ends of the chain is varied in the interval ΔT= [5–45] K. Thus, we have investigated the energy transport rate of three protein lengths for different ΔT, cf. Figure 3b. The energy transport rate does not change significantly for long-length chains. The temperature difference has a considerable influence on energy transport along the N=20 amino acids chain (short-length chain). The energy transport rate reaches about j=120μeV/s at ΔT=10 K. Figure 3c shows a zoomed figure of the energy transport rate, *j*, along the N=150 and N=300 sites chain for different temperature differences, ΔT. Hence, we obtain that we can modulate the energy transport along short protein chains through modulation of the temperature difference between their two ends.

### 3.3. Solvent

Salt concentration has an effective role in the stability and biological function of biomolecules [23,24,25]. Several studies take into account the effect of salt concentration on proteins [26]. Ion-specific effects on proteins and their biological functions were studied previously [27,28,29]. A solvent potential for a system with hydrogen bonds can be introduced through the following term [30]:

(10) $$H_{sol}=-f_{s}\left[\tanh(\frac{u_{i}}{l_{s}})-1\right],$$

where the factor $f_{s}$ denotes the salt concentration and its effect on the system and $l_{s}$ defines the width of the hump that modifies the phonon type potential energy of amino acid residues.

**Figure 4:**
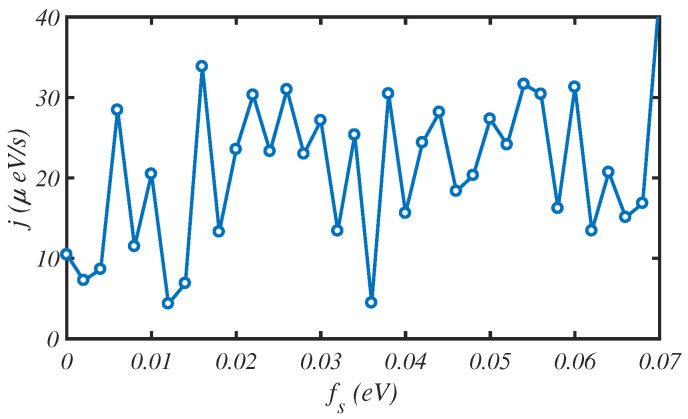
The fluctuation of energy flux, *j*, through a protein chain of N=150 sites, for different values of the solvent parameter, $f_{s}$, at T=300 K.

**Figure 5:**
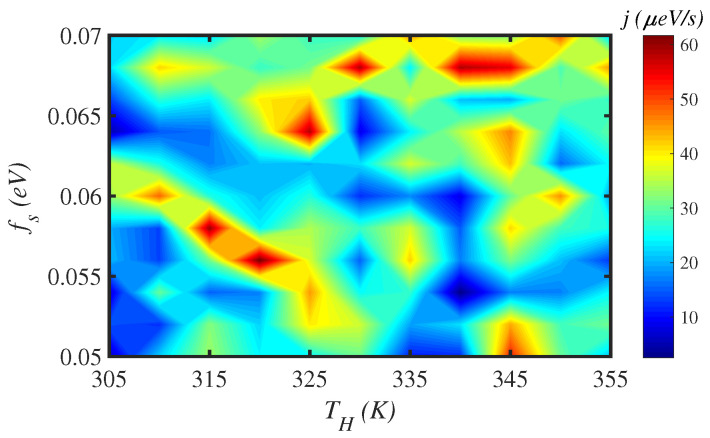
Color map of the variation of energy flux, *j*, through a N=150 site protein chain with respect to the influence of the solvent parameter, $f_{s}$, and of the temperature of hot source, $T_{H}$, when the protein is subjected to a fixed temperature difference, ΔT=5 K, between its two ends.

We have investigated the energy transport rate of a N=150-site protein chain at T=300 K for different salt concentrations, i.e., different values of the $f_{s}$ factor. This is shown in Figure 4. In Figure 5, we change both $f_{s}$ and the temperature of the hot source $T_{H}$ for a fixed temperature difference ΔT=5 K between two ends of chain. We observe that the energy transport rate is sensitive to the salt concentration and to $T_{H}$. The energy flux fluctuates severely as the maximum energy transport rate reaches about j= 60 μeV/s for some values of $f_{s}$.

### 3.4. External Force

**Figure 6:**
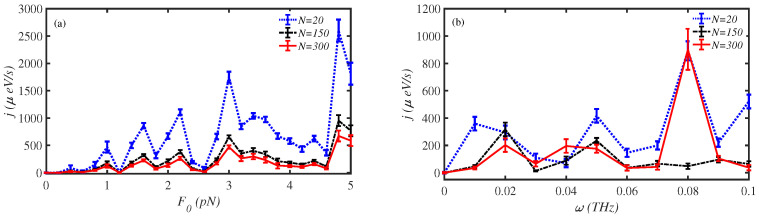
The energy transport rate, *j*, along protein chains of different length, subjected to external mechanical load at T=300 K, (**a**) with respect to the magnitude of the force $F_{0}$ at fixed frequency ω=0.1 THz, (**b**) with respect to the force frequency ω at fixed magnitude $F_{0}=2$ pN.

The external force can be an effective agent on energy flux. It can excite the system through the following Hamiltonian [31,32]:

(11) $$H_{F}=-\delta_{1,i}\left(u_{i}\right)F_{0}\sin\left(\omega t\right),$$

where $F_{0}$ and ω are the external force magnitude and frequency, respectively, while $\delta_{1,i}$ implies that the external force is imposed on the first site. We have considered the influence of magnitude and frequency of external force on the energy flux along protein chains of different lengths, cf. Figure 6a,b.

In Figure 6a, we have fixed the external force frequency at ω=0.1 THz and studied the effect of force magnitude, $F_{0}$, on energy flux. Our calculations show that $F_{0}$ has a considerable effect on energy transport along the protein chain. These results determine that the external force amplifies the energy flux of all chains with different lengths. Energy flux along the short length chain reaches j=3000μeV/s for $F_{0}=4.8$ pN. Even the long-length chains pass energy at about j=500μeV/s for $F_{0}=4.8$ pN. This means that the external force magnitude is among the most effective factors determining energy transport along protein chains. In Figure 6b, we have fixed the external force magnitude at $F_{0}=2$ pN and varied its frequency ω. The frequency ω=0.08 THz is such that it intensifies the energy flux up to about j=1000μeV/s. The remarkable point is that the energy flux along the long-length chain (N=300) intensifies at ω=0.08 THz, too. We conclude that the external force is an appropriate modulator for controlling the energy transport along a protein chain.

**Figure 7:**
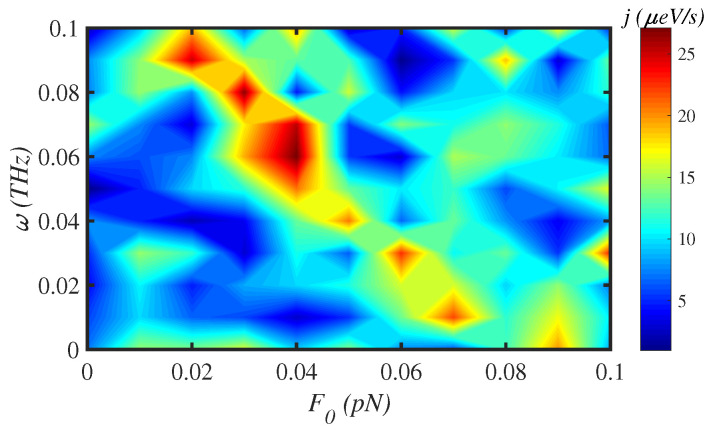
The energy flux *j* map through a N=150 site protein chain under the influence of both the magnitude $F_{0}$ and the frequency ω of the external mechanical force at T=300 K.

**Figure 8:**
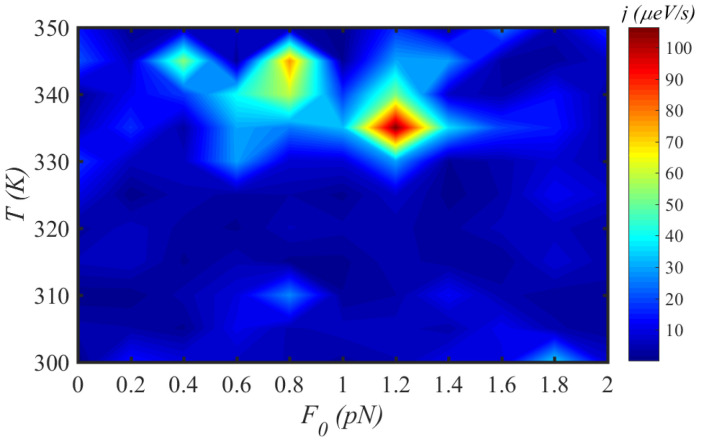
The energy flux *j* map through a N=150 site protein chain under the simultaneous effects of temperature *T* and magnitude $F_{0}$ of the external mechanical load at a fixed force frequency, ω=0.1 THz.

**Figure 9:**
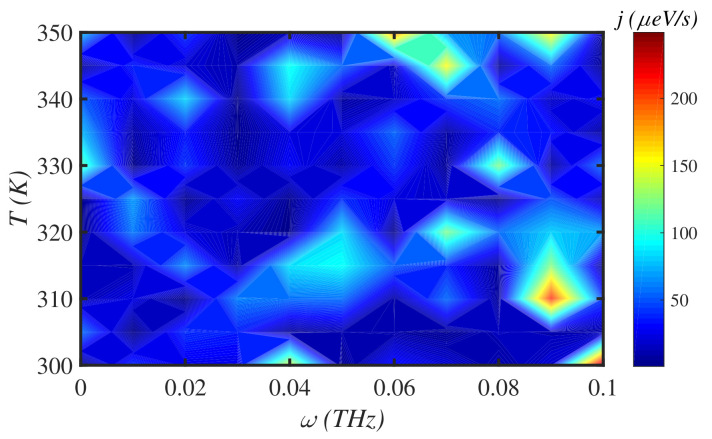
The energy flux *j* map through a N=150 site protein chain under the simultaneous effects of temperature *T* and frequency ω of external mechanical load at a fixed force magnitude, $F_{0}=$ 2 pN.

Figure 7 demonstrates the energy flux along a N=150-site chain, in the low-magnitude force regime, for different drive frequencies, at physiological temperature, T=300 K. There are islands with different colors that determine the sensitivity of energy transport on the external force factors, $F_{0}$ and ω. In the low-magnitude region, the energy flux is maximum for $F_{0}=0.04$ pN and ω=0.06 THz. On the other hand, in Figure 8, we have simultaneously changed the temperature in the interval [300–350] K and the external force magnitude for a fixed frequency, ω=0.1 THz, to investigate the energy flux *j* for a chain of N=150 sites. The energy flux is shown almost constant in the energy colored map. The most distinct colored area for $F_{0}=1.2$ pN and T=335 K determines the maximum energy flux along the system. Finally, in Figure 9, we have fixed the force magnitude at $F_{0}=$ 2 pN and considered the simultaneous effect of force frequency and temperature on the energy flux.

### 3.5. Multifractal Analysis

Predicting and determining the critical behavior has an essential role in studying the future of nonlinear dynamical systems. Most natural systems show self-similar fractal behavior [33,34,35]. Some of the most complicated systems such as DNA and protein chains cannot be characterized using only one fractal dimension. It is necessary to define a fractal dimension spectrum for such systems known as multifractals. For protein chains, multifractal analysis is one of the valuable tools to distinguish their distinct properties. In the current study, we have used multifractal analysis to determine the characteristic parameter values.

We have divided the *d*-dimensional phase space of the system into cubes of size $a^{d}$ utilizing the description of the generalized dimension

(12) $$D_{q}=\frac{1}{q-1}\lim_{a\to 0}\frac{\ln\sum_{i}^{M(a)}P_{i}^{q}}{\ln a},$$

where $P_{i}$ is the probability that the trajectory on the strange attractor visits cube *i* and M(a) is the number of cubes that are not empty [36]. To get a thermodynamic explanation of multifractality, we consider the standard demonstration $\tau \left(q\right)=D_{q}\left(q-1\right)$, where τ(q) defines an analogous free energy. On the other hand, an analogous specific heat is given by the second-order derivative of τ as $C\left(q\right)=-\frac{\partial^{2}\tau }{\partial q^{2}}\approx \tau \left(q+1\right)-2\tau \left(q\right)+\tau \left(q-1\right)$ [37]. The form of C(q) resembles a classical phase transition at a critical point.

**Figure 10:**
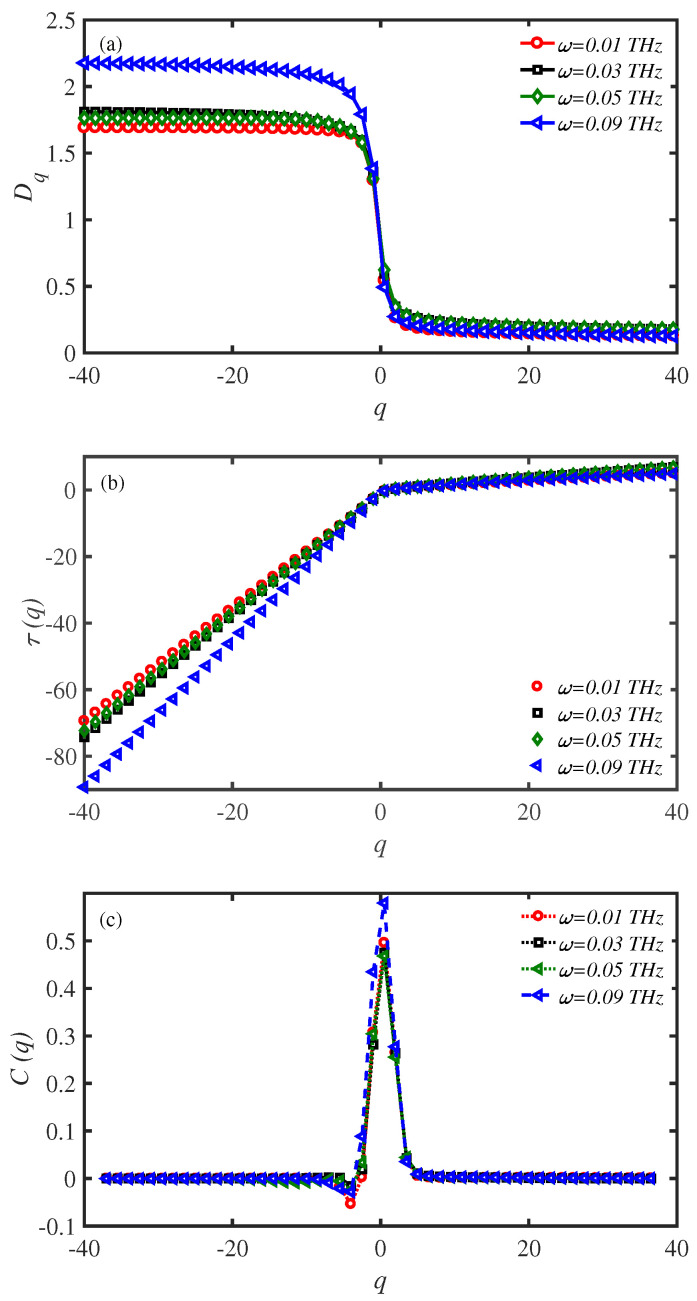
Multifractal analysis of the energy transport rate along a N=150 site protein system, at T=310 K. (**a**) The generalized dimension $D_{q}$, (**b**) the analogous free energy τ(q), and (**c**) the analogous specific heat C(q), for a fixed mechanical load magnitude, $F_{0}=$ 2 pN.

In Figure 9, the force frequency ω=0.09 THz, at T=310 K, is a characteristic value in which the system shows distinct behavior. We have set the system temperature at T=310 K and varied the frequency of the external force to study the multifractal properties of the system (Figure 10a–c). Figure 10a shows the generalized fractal dimensions, $D_{q}$, of the system. The system is multifractal at all frequencies, since $D_{q}<D_{\overset{´}{q}}$ for $\overset{´}{q}>q$. However, the multifractal spectrum of the system at ω=0.09 THz is different than others at negative values of *q*. The value of $\Delta q=D_{q_{max}}-D_{q_{min}}$ is maximum at ω=0.09 THz. The result confirms the distinct behavior of the system at ω=0.09 THz obtained in Figure 9, previously. Figure 10b presents the analogous free energy in multifractal systems, τ(q) [38,39] for different values of force frequency. According to the relation τ(q), it is a straight line for single fractal systems, while it deviates from a straight line for multifractal systems. Figure 10b supports the previous result based on the system multifractality at all force frequencies. This further confirms the multifractality of the system at ω=0.09 THz compared to other values [40]. Figure 10c shows the analogous specific heat, C(q), in multifractal dynamical systems for different values of external force frequencies. C(q) curves have one single main peak that determines the classical (first-order) phase transition at the critical point. C(q) gets a higher maximum peak than others at ω=0.09 THz. This means that the maximum energy flux shows a peak higher than in other cases. This result is confirmed via the $D_{q}$ spectra, which shows that the maximum energy flux has higher dimension spectra.

Generally, the obtained results from multifractal analysis confirm the distinct behavior of the protein system at a characteristic frequency of external force. Such distinct behavior in our system corresponds to maximum energy flux along our protein according to Figure 9. Therefore, multifractal analysis is an appropriate tool to predict and investigate the critical parameter values and confirm the results.

## 4. Discussion

Energy transport in biological systems is a challenging matter from both biological and physical points of view [41]. The redistribution of energy can be used to provide mechanical motion [42,43], mediate allosteric communication [44,45], and drive bioenergetic processes [46]. This energy is either dissipated to generate thermally induced stress for inducing mechanical motion or used to promote catalytic activities. Therefore, an efficient and controlled energy transport is critical for the function of biological motors and nanomachines.

In the current study, we have used an improved Davydov model in a one-dimensional approximation of an isolated chain of hydrogen-bonded peptide groups to study the energy transport in α-helix protein chains. Different factors affect bioenergy transport [47]. Most of the energy transmission in biosystems is diffusive and sensitive to temperature and molecular conformation. Environmental factors such as temperature, pH, concentration, and the presence of denaturants can generate structural transitions in the system and affect energy transport.

We have considered the effect of temperature and temperature difference between two ends of the chain on the energy flux of the system. We have changed the temperature from physiological temperature (T=300 K) up to 350 K. Previous studies have shown that vibrational energy transport along a helical protein system changes from inefficient and ballistic below a critical temperature to diffusive and obviously more efficient above that temperature. This change can be due to the increased flexibility of the helix above this temperature. Structural flexibility improves intramolecular vibrational energy redistribution. It feeds the energy back into several vibrational modes that delocalize over large parts of the structure and transport energy efficiently. Consequently, one can control the energy transport by engineering the temperature or temperature gradient along the system.

On the other hand, as a common cofactor, the presence of salt ions is required for certain biological processes involving proteins. Salt concentration has a significant influence on the conformational behavior of the system [48]. We have also studied the energy flux through the protein chain subjected to an external mechanical force. Biomolecules are often exposed to functionally necessary mechanical pressures, e.g., within muscle fibers, microtubules, and molecular motors, in addition to interacting with other molecules in the cell. The evolution of biomolecules has probably led to optimizing their mechanical behavior to accommodate their biological function [49].

External forces alter the energy landscape of proteins and drive them into functionally significant unfolded or deformed conformations [50]. Different conformations influence the energy transport properties of the protein system. We have obtained that the external mechanical force magnitude is the most influential factor on energy flux through the protein chain. The external mechanical force can enhance the energy transport along the protein up to several hundred times. Therefore, we can accept the external force as a effective controller on energy transport along protein biomolecules.

Finally, we have used multifractal analysis to obtain and predict critical parameters. Multifractal analysis was employed for predicting and confirming the results in other biological systems previously [35,51]. Multifractal analysis was found to help examine critical behaviors and confirm previous results.

## 5. Conclusions

Generally, we found that studying the important factors of energy transport along the protein chain (length, temperature, temperature gradient, solvent concentration, external force) and performing multifractal analysis can help us control it for potential biological and nanotechnological applications. The results determine that the energy transport rate is high in the short protein chains. The average energy fluctuations for a short-length amino acid chain are greater than those of longer chains. On the other hand, the external force is the most effective factor on energy transport in protein molecules. In the future, we will try to investigate the effect of electromagnetic fields on energy transport along protein chains and study charge and energy transport simultaneously. The obtained results can help us design biological molecular motors based on protein chains with improved functionality.

## Data Availability

Data available upon reasonable request.

## Abbreviations

The following abbreviations are used in this manuscript:

ATP	adenosine triphosphate
ADP	adenosine diphosphate
